# Producing Stem Cell-Based Transplants for Future Therapeutic Purposes

**DOI:** 10.1155/2017/9592302

**Published:** 2017-12-11

**Authors:** Iván Velasco, Tilo Kunath, Verónica Ramos-Mejía, Marco A. Velasco-Velázquez

**Affiliations:** ^1^Instituto de Fisiología Celular-Neurociencias, Universidad Nacional Autónoma de México, 04510 Ciudad de México, Mexico; ^2^Laboratorio de Reprogramación Celular del IFC-UNAM, Instituto Nacional de Neurología y Neurocirugía “Manuel Velasco Suárez”, 14269 Ciudad de México, Mexico; ^3^University of Edinburgh, Edinburgh EH16 4UU, UK; ^4^Centre for Genomics and Oncological Research (GENYO), 18016 Granada, Spain; ^5^Departamento de Farmacología, Facultad de Medicina, Universidad Nacional Autónoma de México, 04510 Ciudad de México, Mexico; ^6^Unidad Periférica de Investigación en Biomedicina Translacional, ISSSTE C.M.N. 20 de Noviembre, Facultad de Medicina, Universidad Nacional Autónoma de México, 03100 Ciudad de México, Mexico

Stem cells have been used in the clinic for very specific pathologies, but research has been conducted to develop new therapies for a wider range of human diseases. Currently, there is a great need for step-by-step investigations that consider all aspects related to cell therapy in disease-specific contexts. The objective of this special issue is to bring together timely reviews as well as original papers on different aspects related to the use of stem cells or their progeny in transplantation procedures that can positively modify degenerating or accidentally damaged tissues and organs. A considerable number of manuscripts were submitted, and after peer review, eight papers were published. The topics of these papers are diverse, including the generation of immortalized cells with inducible systems, the biological behavior of transit-amplifying cells, the expansion capacity of mesenchymal cells over hematopoietic cells, and the influence of growth factors in the maintenance of a differentiated phenotype. Regarding transplantation procedures, the nonimmunogenic intrauterine grafting of muscle cells and cell replacement therapies for retinal degenerative conditions are reviewed. Evaluation scales to assess the effects of therapies including cells and biocompatible support agents after damage to the spinal cord are also discussed. Grafting of pluripotent stem cell products poses a risk for the development of teratomas; thus, several factors were studied to identify strategies to eliminate tumorigenic cells. We think that these papers cover several of the areas that are important for the proper handling of stem cells to become therapeutically relevant and suggest improvements on the evaluation after grafting of biological materials, which in the long term might lead to new therapeutic interventions.

The paper by K. An et al. presents a potentially relevant tool for the analysis of stem-cell based therapies in the field of pain therapy. First, they immortalized rat bone marrow mesenchymal stromal cells (BMSCs) by introducing the human telomerase reverse transcriptase (hTERT). Then, they introduced a tetracycline-inducible lentivirus delivery system to express the rat GAL gene into the immortalized rat BMSCs. The authors further characterized the phenotypic properties of these cells and confirmed their ability to produce GAL in a doxycycline-controlled manner. Finally, the authors advise that further studies are necessary to rule-out the possibility of transformation after long-term expansion of hTERT-BMSCs and to eliminate the leakiness of this inducible system.

The field of in utero transplantation (IUT) has, until recently, been focused on the hematopoietic system. The review by N. Chowdhury and A. Asakura comprehensively describes the literature on in utero stem cell transplantation, covering hematopoietic applications as well as multiple other cell types and organ systems. The detection of genetic disorders in utero is now possible due to advances in cell-free fetal DNA diagnostics in maternal blood. This provides an opportunity for intervention and the application of in utero stem cell transplantation therapies. The authors explore the emerging area of IUTs for muscle disorders, in particular muscular dystrophies. Attempts to treat children with muscular dystrophy with muscle stem cell transplantations have failed partially due to the sheer number of cells needed to treat a patient. One solution outlined in detail by the authors is to treat the fetus in utero by transplantation of muscle stem cells.

Human adipose-derived stem cells (hASCs) are a source of differentiated cells that could be employed in regenerative medicine. A. E. Mortimer et al. examine the role of different stimuli in the *in vitro* maintenance of Schwann-like cells produced from hASCs. Authors found that basic fibroblast growth factor (FGF-2) was essential for the maintenance of the Schwann-like phenotype. In contrast, differentiation was partially preserved in absence of glial growth factor 2 (GGF-2), platelet-derived growth factor (PDGF), or forskolin. The lack of forskolin in the culture medium reduced mRNA and protein levels of brain-derived neurotrophic factor (BDNF), suggesting that an elevated cAMP concentration and the consequent CREB activation are required for BDNF expression. Finally, they identified that GGF-2 alone supports the expression of Krox20, a transcription factor indispensable for myelination. The evidence presented in this paper indicates that maintenance of the Schwann-like cells requires the synergistic activation of different signaling pathways.

The paper by O. Gordeeva and S. Khaydukov explores the differences in the differentiation potential between embryonic stem (ES) cells and embryonal carcinoma (EC) cells in a retinoic acid differentiation protocol. During differentiation, the ES cells lost pluripotency markers, such as *Oct4* and *Nanog*, more readily than EC cells and then gained some, but not all, differentiation markers with faster kinetics. In particular, *Activin A*, *BMP4*, *TGFβ1*, and *Nodal* were more highly expressed in differentiating ES cells when compared to EC cells. The addition of these TGF*β* superfamily members as recombinant proteins to the differentiation conditions further enhanced loss of pluripotent markers and increase commitment to differentiated lineages. This also further reduced the tumorigenicity in teratoma assays. This work shows that activation of TGF*β* signaling in ES cells can decrease their tumorigenicity and increase their safety profile for cell-based therapies.

The review by E. Rangel-Huerta and E. Maldonado describes the different biological properties of transit amplifying cells (TACs), early intermediates in tissue regeneration. With special focus on epidermal skin studies, they discuss important aspects of TACs such as gene expression profiles, division rates, asymmetric cell division, and environmental cues that may regulate their behavior. The authors propose that understanding TACs' biology is a key issue for their implementation in regenerative medicine.

Transplantation of stem cell-derived retinal pigmented epithelium (RPE) has already began in human clinical trials. However, there are no universally agreed upon animal models to assess the efficacy and safety of these therapies to treat these retinal degenerative conditions. The review by T.-C. Lin et al. systematically surveys all the animal models in use for this type of stem cell-based therapy. They cover mouse, rat, rabbit, dog, cat, pig, and nonhuman primate models of preclinical retinal degeneration. Since the therapies are using human cells, they discuss various methods to deal with immunosuppression in the animal models. A particularly novel one is the description of the crossing of immunodeficient nude rats (*Foxn1*-null) to the RCS rat that suffers from a progressive form of retinal degeneration. Although the larger animal models are useful for refining the surgical techniques, the rodent studies have the benefit of larger animal numbers and genetic manipulation to more closely model the human condition. A consensus on the most informative models of efficacy and safety for testing RPE cell products requires further exploration.

Spinal cord injury (SCI) is a common lesion in accidents, which cause disability in people; currently, there are no efficient therapeutic interventions in the clinic. The review paper by W. R. Kim et al. relates to the importance of having grading scales that are both easy to use and reproducible to assess the damage, as well as the recovery, on experimental animals subjected to SCI. The damage to this part of the central nervous system can have severe and long-lasting affections to the anatomy and the function of motor aspects that are greatly influenced by the extension and the anteroposterior position of the injury. After describing the most widely used animal models for SCI, a summary of the beneficial effects of stem cell grafting, either alone or combined with biomaterials, is presented. This section clearly shows that several types of cells, trophic factors, and polymers can all contribute to the correction of disturbed functions. The final part discusses the different scales to measure spinal cord function for rodents and nonhuman primates, highlighting its advantages and limitations.

Mesenchymal stromal cells are found in the bone marrow and contribute to the microenvironment that preserves hematopoietic stem cells *in vivo*. Mesenchymal stem/stromal cells (MSCs) have also been isolated from other sources such as adipose tissue, umbilical cord, and placenta. Hematopoietic stem and progenitor cells (HPCs) can be expanded or differentiated by coincubation with bone marrow MSCs *in vitro*. Perinatal sources of MSCs such as the umbilical cord and placenta are more accessible than the bone marrow aspirates. In the paper by G. R. Fajardo-Orduña et al., the ability of cord blood and placenta MSCs to expand HSCs was compared to the standard bone marrow MSCs. After immunophenotypic and functional characterization of the three types of MSCs, the expansion potential for primitive HSCs was tested without significant differences between the different MSCs. Also, the induction of myeloid colony forming units was very similar for bone marrow, umbilical cord, and placenta. These results suggest that MSCs from different origins might be used in the expansion of HSCs aimed to transplantation.

